# ViMIC: a database of human disease-related virus mutations, integration sites and *cis*-effects

**DOI:** 10.1093/nar/gkab779

**Published:** 2021-09-09

**Authors:** Ying Wang, Yuantao Tong, Zeyu Zhang, Rongbin Zheng, Danqi Huang, Jinxuan Yang, Hui Zong, Fanglin Tan, Yujia Xie, Honglian Huang, Xiaoyan Zhang

**Affiliations:** Research Center for Translational Medicine, Shanghai East Hospital, School of Life Sciences and Technology, Tongji University, Shanghai 200092, China; Department of Laboratory Medicine, Shanghai Eastern Hepatobiliary Surgery Hospital, Shanghai 200438, China; Research Center for Translational Medicine, Shanghai East Hospital, School of Life Sciences and Technology, Tongji University, Shanghai 200092, China; Research Center for Translational Medicine, Shanghai East Hospital, School of Life Sciences and Technology, Tongji University, Shanghai 200092, China; Research Center for Translational Medicine, Shanghai East Hospital, School of Life Sciences and Technology, Tongji University, Shanghai 200092, China; Research Center for Translational Medicine, Shanghai East Hospital, School of Life Sciences and Technology, Tongji University, Shanghai 200092, China; Research Center for Translational Medicine, Shanghai East Hospital, School of Life Sciences and Technology, Tongji University, Shanghai 200092, China; Research Center for Translational Medicine, Shanghai East Hospital, School of Life Sciences and Technology, Tongji University, Shanghai 200092, China; Research Center for Translational Medicine, Shanghai East Hospital, School of Life Sciences and Technology, Tongji University, Shanghai 200092, China; Research Center for Translational Medicine, Shanghai East Hospital, School of Life Sciences and Technology, Tongji University, Shanghai 200092, China; Research Center for Translational Medicine, Shanghai East Hospital, School of Life Sciences and Technology, Tongji University, Shanghai 200092, China; Research Center for Translational Medicine, Shanghai East Hospital, School of Life Sciences and Technology, Tongji University, Shanghai 200092, China

## Abstract

Molecular mechanisms of virus-related diseases involve multiple factors, including viral mutation accumulation and integration of a viral genome into the host DNA. With increasing attention being paid to virus-mediated pathogenesis and the development of many useful technologies to identify virus mutations (VMs) and viral integration sites (VISs), much research on these topics is available in PubMed. However, knowledge of VMs and VISs is widely scattered in numerous published papers which lack standardization, integration and curation. To address these challenges, we built a pilot database of human disease-related **Vi**rus **M**utations, **I**ntegration sites and **C**is-effects (ViMIC), which specializes in three features: virus mutation sites, viral integration sites and target genes. In total, the ViMIC provides information on 31 712 VMs entries, 105 624 VISs, 16 310 viral target genes and 1 110 015 virus sequences of eight viruses in 77 human diseases obtained from the public domain. Furthermore, in ViMIC users are allowed to explore the cis-effects of virus-host interactions by surveying 78 histone modifications, binding of 1358 transcription regulators and chromatin accessibility on these VISs. We believe ViMIC will become a valuable resource for the virus research community. The database is available at http://bmtongji.cn/ViMIC/index.php.

## INTRODUCTION

Viruses are of substantial interest due to the seriousness of their associated diseases in humans. The mechanism of viral pathogenesis is complicated, as viruses interact with the host genome on multiple levels (1,2). For example, studies revealed that a virus may exert *cis*-effects by integrating its genome into human chromosomes (3–6). Mutations in viral regions or genes may also contribute to an increased risk for disease progression and drug resistance (7–9). Some virus mutations (VMs) may play an important role in promoting the occurrence of diseases by increasing or decreasing the incidence of viral DNA integration into host cells (10–13). Moreover, alterations in epigenetics may also be induced by the viral protein to promote activation or inhibition of target genes (14–17).

At present, millions of virus studies can be found in PubMed. Such studies have generated vast amounts of data relevant to VMs and viral integration sites (VISs) covering different types of viruses and human diseases. Meanwhile, millions of virus sequence data deposited in public databases may be used to discover feature patterns related to diseases. Domain knowledge that can be used to improve the understanding of virus cis-effects, for instance, gene expression and transcription cis-regulatory information, has been deposited in freely accessible databases, such as Cistrome Data Browser (18,19) and the Gene Expression Omnibus (GEO) (20). However, well-curated information and data are distributed in separate public domains that lack integration and standardization. Therefore, it is challenging for researchers to quickly browse, visualize and re-use such knowledge for their studies of virushost interactions. Several databases, such as the Viral Integration Site DataBase (VISDB) (21), Dr.VIS (22), the Retrovirus Integration Database (RID) (23) and the ISDB (24) have been developed. However, to our knowledge, these databases focused on virus integration, and none of existing databases or webservers are designed for systematically evaluating VMs and functionally annotating of VISs. An understanding of viral pathogenic mutations and extraction of feature patterns mined from virus sequence data will improve effective surveillance and early detection for high-risk virus-infected diseases (25,26). Significant viral mutations/regions for affecting integration reactions (10,11,13,27) and the comparative analysis of virus integration/mutation landscapes at cfDNA level and paired tissues (28) have been reported. Previous studies have also demonstrated that transcriptional regulators contribute to the control of viral replication and regulate critical cis-elements in a virus (29,30).

Therefore, to overcome the challenges mentioned above, we present a database of human disease-related **Vi**rus **M**utations, **I**ntegration sites and *cis*-effects (ViMIC). ViMIC is built to fill such gaps from the aspects of virus mutation, integration, and *cis*-effect of host. The unique features of our database over other databases include: ViMIC is the first database to provide VMs and virus sequence data annotations retrieved from a large collection of public domains; and ViMIC is the first to visualize VMs harbored in VIS fragments and integrate VIS data with cistrome resources to help researchers who are interested in gene regulation mechanisms to systematically investigate the functional annotation of VISs in the host genome. ViMIC mainly covers three special features: virus mutation sites, viral integration sites and target genes. Firstly, the database facilitates the search for VM annotations and quick browsing for statistics on virus sequences. Secondly, the database can help users determine whether the integration site of viral fragments harbor mutations or whether the VIS integrated into the host genome is in a functional element distinguished by histone modification, transcription factor binding or chromatin accessibility. Thirdly, the database provides information on host target genes affected by viral insertion or by virus gene/protein/region, and on the correlation between gene expression levels and the fraction of infiltrating immune cells in human diseases.

## CONSTRUCTION AND CONTENTS

**Figure 1.**
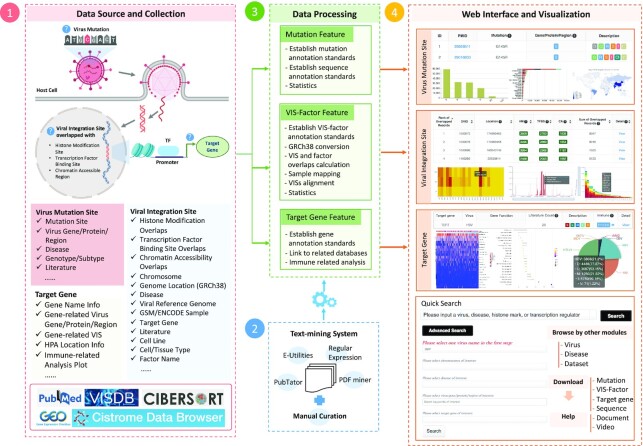
A schematic overview of the ViMIC database. ViMIC collects publicly available virus mutations (VMs), viral integration sites (VISs), cistrome factors and target genes information from multiple public resources, including PubMed, Cistrome Data Browser, VISDB and GEO. ViMIC uses text mining to extract the virus-related bioconcepts from published citations, followed by a manual curation to ensure annotation accuracy. The data processing layer is responsible for specific tasks, including establishing annotation standards stored in a MySQL relationship database, performing VIS mapping to the reference genome, calculating overlaps between VIS and cistrome factors, analysing the correlation between gene expression and infiltrating immune cell fractions and generating statistical plots. The ViMIC interface was designed with three main features: virus mutation sites, viral integration sites and target genes. ViMIC provides three ways to query and explore data: by keyword search, quick search engine or an advanced search menu. In addition, ViMIC includes other modules, labelled as Virus, Disease, Dataset, Download and Help that allow users to better explore the collected data.

A schematic overview of the ViMIC data collection, text-mining system, data processing and web interface is summarized in Figure 1.

### Data sources

The major sources of data in ViMIC were derived from the published literature, the Cistrome Data Browser (18,19), VISDB (21), NCBI Nucleotide and the GEO database (20). Viral annotation information was retrieved from a large collection of published literature through our text-mining system, which was followed by a manual curation process. Virus sequence information was obtained in batches from public repositories, mainly from the NBCI Nucleotide Database. Viral integration sites were retrieved from published literature and VISDB. The human transcription regulator binding, histone modification, and chromatin accessibility data were obtained from the Cistrome Data Browser. Gene expression datasets of virus-infected diseases were collected from GEO as well as the Cancer Genome Atlas (TCGA).

### Text mining and data processing

The Entrez Programming Utilities (E-Utilities) tool was used to build a set of full names and abbreviations of virus keywords into a fixed URL syntax to search and retrieve virus-related articles from PubMed. A Python script was run to filter the articles by matching keywords (e.g. mutation) as regular expressions. Full-text articles from PMC and abstracts from PubMed (BioC-XML format) with annotations of biomedical concepts in the Bioconepts2pubtatorcentral file were downloaded from PubTatorCentral (31), and this file was then used to extract biomedical entities such as genes, mutations, diseases and chemicals. A Python script was developed to automate the entity recognition task for those articles without PubTator annotation. The procedure included literature downloading, conversion of PDF documents to plain text files (PDFMiner library), and noise filtering and information extraction for sentences containing mutations. Mutation entries were automatically extracted from the PubMed and PubTator annotation files (32), followed by a manual curation to ensure the data quality of our database.

For the virus sequence module, GenBank id, virus gene, genotype, geographical region and disease information was extracted from the NCBI Nucleotide database for standardized annotation and statistics. For the VIS-VM module, virus integration fragments were collected from publicly available literature. Since the information of some collected VISs may be incomplete, we only focused on those VISs where both breakpoint locations and the viral reference genome were provided. The multiple sequence alignment tool CLUSTALW (33) was then used to identify mutations harbored in each VIS. The percentage of base consistency between collected VIS sequences and a set of reference sequences was checked to evaluate the influence of reference genome choice on the result. The percentage consistency was 93.62% when using the reference given in the original literature, while the percentage consistency was only 79.43% if all references derived from the literature and the NCBI database were combined. This indicated that it was feasible to apply literature-chosen references in the VIS-VM module. Moreover, for each target gene, a correlation analysis was conducted between target gene expressions and fractions of infiltrating immune cells estimated by Cell-type Identification By Estimating Relative Subsets Of RNA Transcripts (CIBERSORT) (34).

### VIS-cistrome data (factor) overlapping analysis

To construct features for exploring the functional integration of a VIS (VIS-factors), the overlaps were calculated between the VIS and cistromes. The concept of ‘cistrome’ has been widely used in the gene regulation field (18,19,35). As these studies state, a cistrome refers to ‘the set of cis-acting targets of a *trans*-acting factor on a genome-wide scale, also known as the *in vivo* genome-wide location of transcription factor binding-sites or histone modifications’. Therefore, the host gene expression may be influenced if the virus integrates into the Cistrome. Cistrome DB collects human and mouse ChIP-seq, DNase-seq and ATAC-seq data from publicly available resources. In our current study, Cistrome DB data was applied as cistrome factor information to integrate with data from a virus integration site and assist users to explore the cis-effect of VIS. The procedure included four steps:

Firstly, the viral integration sites in the human genome were derived from VISDB (76 264 curated VISs) and from the PubMed literature (29 360 VISs, 48 articles) that reported disease-related viral integration events in the human genome. The collected VISs were processed into BED format, which was then converted into human reference genome coordinates in GRCh38 by the LiftOver if a previous genome version was used. Secondly, the ChIP-seq peaks of human transcription factor and histone modifications were downloaded as well as the DNase-seq and ATAC-seq peaks from the Cistrome Data Browser. This information was then compressed into bgzipped (.gz) files (a broad peak file using all peaks and a narrow peak file using 5-fold-enriched peaks). Thirdly, a GIGGLE (genome search engine) (36) index was built using the compressed peak files, and GIGGLE was used to identify and search the significance of shared genomic loci (overlap) between the VIS and three genome interval files (histone modification site, transcription factor binding site and chromatin accessible region). Finally, the GSMID and biological resource information was incorporated from the Cistrome Data Browser into the VIS-cistrome entry (overlap > 0). If a VIS overlapped with cistrome factor-related GSM samples, it would indicate that the virus integration site is a potentially functional DNA element that can influence gene expression in the human.

The location used in ViMIC is according to the coordinate in the human genome of VIS declared in the original articles. There are two forms of VIS location, one is VIS with a single site, and the other is VIS with both two positions. In our data, the single site accounts for nearly 94%. VISs with two positions were generally classified into two categories. Case 1: the deletion of human fragments during the integration event (i.e. Jacques Thomas *et al.*) (37); Case 2: due to the experimental method/design, two viral insertion positions were provided in the literature (i.e. Song Cao *et al.*, Maria Artesi *et al.*) (38,39). We compared overlaps between VIS with single site and two positions (Supplementary Figure S1A), and visualized the overlap difference between them (Supplementary Figure S1B). Supplementary Figure S1A showed that the overlaps between VISs and Cistrome data were different in the single site cases, demonstrating the potential enrichment of cistrome factors on the specific virus integration segment. We also observed that there was an increased trend when the distance of two positions (length) was over 1 kb. Supplementary Figure S1B showed that there is an obvious difference at the position of 10^1.6^ (40 bp) for all VISs with two positions, which indicated that two-point VIS with length <40 bp would have similar overlap calculation result with the single site. Therefore, for each VIS with two points, the overlapping calculation results of single start site and two positions will be both displayed. Moreover, the classification case of VISs with two positions based on literature (case 1 or case 2) and the length (the distance of two positions) were also added in the web interface for those VIS with two positions.

### Database contents

ViMIC is characterized by a comprehensive curation of VMs, functional annotations of VISs in the host genome, and target genes. ViMIC provides three features, as shown in Figure 1.

#### Feature I—virus mutation site

The ‘Virus Mutation Site’ feature provides two modules for users: the VM annotation module and the Sequence module. The VM annotation module includes mutation site, mutation level, virus gene/protein/region, virus-associated disease, genotype/subtype, and literature evidence. A description column indicates more detailed information for each mutation site using the following tags: (i) D: drugs mentioned in the literature; (ii) G: genotypes/subtypes mentioned in the literature; (iii) R: reference genomes mentioned in the literature; (iv) S: viral sequences mentioned in the literature; (v) I: the VM is related to immune escape; (vi) Cl: clinical information available in the literature and (vii) C: geographical distribution of the source mentioned in the literature. The virus sequence module provides the curated information and virus sequence data statistics including a map of viral sequence source distribution and statistics of virus sequence in the viral gene, genotype and countries. Adenovirus-associated virus type 2 (AAV2) was excluded in this feature because there is no pathogenic mutation information from current studies.

#### Feature II—viral integration site

The second feature is the VIS-cistrome factor overlap calculation, which was specifically designed to explore whether the VIS is involved in a functional region in the host genome. For each VIS, if there are overlaps between the VIS and the genomic peaks of the cistrome factors (sum of overlaps > 0), the detailed results are presented, including the overlap number and annotation of each cistrome sample (GSMID, cell line, cell type, tissue type and factor name). Furthermore, all the VIS-cistrome factor overlapped records on human chromosomes are summarised using heatmap and histogram plots, and the top-ranked cistrome factors ordered by the overlap number in each chromosome using stacked bar plots. Thus, users can view the overlap distribution between the VIS and cistrome factors in the human genome. In the current version of the VIS-VM module, VISs of three viruses including hepatitis B virus (HBV), human papillomavirus virus (HPV) and AAV2, with precise breakpoint coordinates and the reference genome in the integration literature, were used to map and visualize whether mutation occurred in VISs.

#### Feature III—target gene

This feature provides information for the target genes affected by viral insertion or by the virus gene/protein/region. We have systematically annotated the target gene information, including official gene symbols, official full names, gene aliases, transcripts, gene types, gene location information, gene functions, gene IDs and drug information. For each gene, if there were available gene expression data for patients with the virus-related disease, we further analysed the relationship between its expression and immune cell infiltration and represented it by heatmap and lollipop plots.

In the current version (as of 21 May 2021), the database contains 8 viruses, including HBV, HPV, Epstein-Barr virus (EBV), AAV2, Merkel cell polyomavirus (MCV), human immunodeficiency virus (HIV), human T-cell lymphotropic virus type-1 (HTLV1) and xenotropic murine leukaemia virus-related virus (XMRV), as well as 31 712 VMs entries, 105 624 VISs (95 280 VISs, sum overlaps > 0) entries, 17 778 436 overlapping entries between VISs and Cistromes, 77 virus-associated diseases (25 public gene expression datasets performing immune analysis), 16 310 target genes, 1 110 015 virus sequence information records and 127 datasets with clinical information. The detailed statistics for ViMIC data are listed in Table 1. As viral integration into host chromosomes plays a key role in viral infection and tumorigenesis, in the current version, we used these eight integration-related viruses to build a database which relates to a variety of major human diseases including cancer (40–43).

**Table 1.** Statistics of the ViMIC data

Data type	Count	Description
Virus	8	Including 5 DNA viruses and 3 RNA viruses.
Virus Mutation (VM)	31 712	Curated mutation information entries.
Virus Sequence	1 110 015	Curated virus sequence information entries.
Histone Mark	78	Histone Mark types overlapped with VISs in ViMIC.
Transcription Regulator	1358	Transcription regulator types overlapped with VISs in ViMIC.
Viral Integration Site (VIS) (Sum Overlaps > 0)	95 280	VISs that overlaps with cistrome data.
VIS-Histone Modification (HM) Overlaps > 0	88 393	VISs that overlaps with cistrome HM data.
VIS-Transcription Factor Binding Site (TFBS) Overlaps > 0	71 668	VISs that overlaps with cistrome TFBS data.
VIS-Chromatin Accessibility (CA) Overlaps > 0	39 127	VISs that overlaps with cistrome CA data.
Virus Related Disease	77	Disease types which associated with the 8 ViMIC viruses.
Target Gene	16 310	Curated target genes affected by viral genome insertion or virus gene/protein/region regulation.
Literature	2539	Count of the literature related to virus mutation and integration.
Clinical Annotation and Data	127	Clinical characteristics and virus sequencing data which are available in literatures.

All of the annotation and analysis results are deposited and managed in a MySQL relational database on a Linux server. More detailed information on the VMs, VISs, and target genes is crosslinked to the related resources including PubMed, VISDB, ENCODE, NCBI GenBank (44), Wikipedia, DrugBank (45), NCBI Gene database (46), Human Protein Atlas (HPA), HUGO Gene Nomenclature Committee (HGNC) (47), Vertebrate Genome Annotation (VEGA) (48), Online Mendelian Inheritance in Man (OMIM) (49), STRING (50), Rfam (51), miRbase (52), lncRNASNP2 (53), GEO, TCGA, ESEMBL (54), International Cancer Genome Consortium (ICGC) and the European Genome-phenome Archive (EGA) (55).

### Database utility

#### Data query

ViMIC allows users to search data of interest in three ways. Firstly, a quick search is available on the homepage. Users can input a keyword of interest including a virus name, disease, histone mark, transcription regulator or virus gene/protein/region. Secondly, there is a search engine at the top-right corner of each table in the web interface, where users can enter any keywords of interest. Thirdly, an advanced search is also available on the home page for sophisticated searches by selecting a virus name of interest, and searching by human chromosome, disease, virus gene/protein/region or target gene. Each search produces tables of matched entries. Users can then view the detailed information by clicking on the hyperlink or the ‘View’ button in the table.

#### Web interface and data visualization

A web interface was designed to provide user-friendly access to browse, search, analyse, visualize and download data. The database provides an interface for each virus with a summary and links to three ViMIC features to help users quickly check their hypothesis regarding VMs, VIS-cistrome factor overlaps or viral target genes.

Firstly, the ‘Virus Mutation Site’ feature presents a catalogue of links to virus genes proteins/ regions and diseases on the homepage of selected virus. In the ‘Mutation’ module, users can view a particular VM labelled with literature PMID, virus gene/protein/region and description. Moreover, users can click the ‘Sequence’ button to browse and download the curated sequence annotation of the selected virus. The detailed virus mutation page is composed of mutation annotations and evidence from the literature.

Secondly, the ‘Viral Integration Site’ feature provides the sum of VIS-cistrome factor counts and three VIS-cistrome factors overlap counts in each human chromosome, as well as two browse data menus for users to view overlapped entries by chromosomes or factors on the homepage of selected virus. Users can browse VIS information by clicking the ‘View’ button in the table as well as by selecting a chromosome or cistrome factor in the browse data menu. ViMIC also developed a function on the web page that allows users to upload a list of integration sites and query them in our database, or users can upload their own VISs location information to the ViMIC and will be notified by email once the job is completed. Moreover, ViMIC designs a VIS-VM module of selected virus to visualize mutations observed in VIS fragments and a search module to show overlap data according to options of a user's interest including disease, biological source (cell line, cell type and tissue type). In addition, ViMIC provides a heatmap of three VIS-cistrome factor overlap counts on each chromosome and two stacked barplots of top-ranked cistrome factors to help users have a better understanding of the distribution of VIS-cistrome factor overlap in the human genome.

Third, ‘Target Gene’ feature provides the curated target genes affected by viral genome insertion or by virus gene/region/protein. Users can browse the detailed gene information, related VISs/VMs and the correlation analysis results between gene expression levels and the proportions of 22 types of infiltrating immune cells in virus associated human diseases.

ViMIC also includes virus, disease, dataset, download and help modules on the homepage, which allows users to be quickly redirected to a particular feature of users’ interest. In addition, users can browse datasets for which clinical characteristics and virus sequencing data are available in the literature in our dataset module. To make the data presentation more intuitive, the statistical plots and tables are also shown on the home page of each module.

#### Case exploration

In this article, we have taken HBV as an example, as it is a viral infection that can cause death in patients, even in anti-viral drug users.

##### Case 1: visualization of the virus mutation site

**Figure 2.**
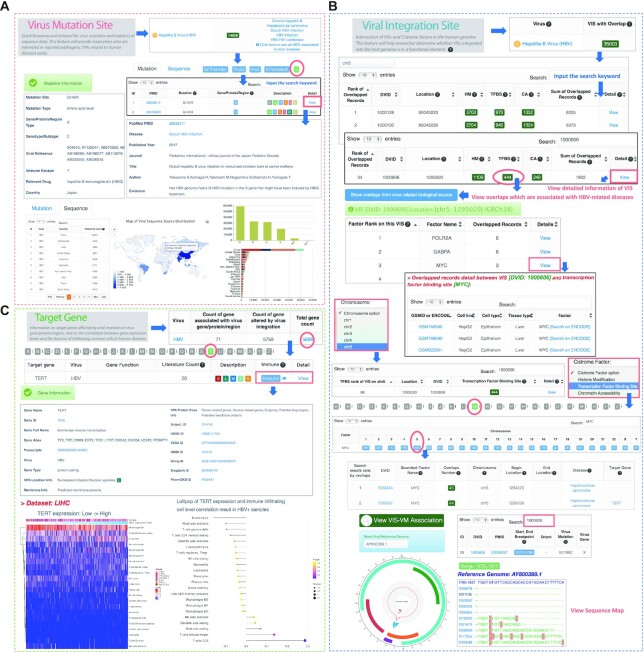
Screenshot depicting an example exploration of the ViMIC database. (**A**) HBV (Hepatitis B virus) is used as an example. The user can access a page on ViMIC containing brief mutation information on HBV by clicking HBV VM Count. The user can input a keyword through the quick search engine and enter the detailed mutation information page to view the mutation site or click the ‘Sequence’ button to quickly browse statistics on virus genome sequence information derived from different regions worldwide and download the curated data information table. (**B**) If the user was interested in acquiring a detailed factor list for VIS entry ‘1000606’ on chromosome 5 in HBV, ViMIC will show the overlaps result of three cistrome factors after the user clicks the ‘chr5’ and searches the entry of ‘1000606’. If the user was interested in the ‘444’ overlap number within the ‘1000606’ and ‘TFBS’ entry, ViMIC will then return the ranking of all transcriptional regulators that have overlaps with this VIS. By clicking the ‘View’ button of MYC, ViMIC will generate a MYC-related table including the GSMID/ENCODE ID, cell line, cell type, tissue type and factor name. On the HBV VIS-cistrome factor homepage, the user can select the chromosome menu to view the overlap distribution of three factors and virus integration fragments on a specific chromosome (e.g. chr5). By selecting the factor menu, the user can explore more overlap statistics and distribution information for a specific transcription factor (e.g., MYC). The user can click the ‘View VIS-VM Association’ button to browse the enrichment of mutations in VISs. By selecting a reference genome (e.g. AY800389.1) and search a DVID, ViMIC will return a sequence alignment result (table and map) showing the distribution of mutations harbored in VISs (e.g. 1000606). (**C**) ViMIC provides the ‘Target Gene’ feature for the curated target genes affected by viral genome insertion or by virus gene/protein/region. If the user was interested in the VIS 1000606 target gene TERT in HBV, ViMIC will show TERT gene information, inserted VISs reported in the literature, and the correlation between gene expression levels and the fraction of infiltrating immune cells in HBV related human disease on the detailed gene information page.

Users can click the green button with VM number of HBV in the virus mutation overview page, as shown in Figure 2A. ViMIC will then return a table containing the manually curated information for the HBV mutation sites. Users can filter the mutation by gene/protein/region or disease, and then enter the keyword in the search box on the top-right of the table and click the ‘View’ button to explore the detailed information of searched mutation, including mutation level, virus gene/protein/region, genotype, as well as evidence mentioned in literatures. Moreover, Users can click the ‘Sequence’ button to browse statistics on the HBV genome sequences derived from different regions worldwide. The information would help users get the advances in research on virus mutation to quickly target mutations of interest.

##### Case 2: visualization of the viral integration site overlapped with cistrome factors

If users wish to acquire a detailed factor list for one VIS entry, they can follow the step in Figure 2B. We take the entry of ‘1000606’ on chromosome 5 in HBV as an example. After users click the ‘chr5’ and search the entry of ‘1000606’, ViMIC shows the overlaps result of three cistrome factors. By clicking the green button with number at ‘TFBS’ (Transcription factor binding site) column and filtering the biological source, ViMIC shows the ranking of 16 transcriptional regulators that have overlaps with this VIS. In the detailed page of ‘1000606’, we found that Ding *et al.* identified the recurrent HBV insertion nearby the telomerase reverse transcriptase (TERT) gene (56). In ViMIC, users can view cistrome samples by clicking the ‘View’ button of the transcription factor MYC and can find that three sample shows an overlap between ‘1000606’ and MYC at the position on chromosome 5 (coordinate range: 1295020, hg38). Previous studies have indicated that HBV integration sites can cause changes in cancer driver genes, including TERT and MYC (57). In HepG2, it also proved that the expression of TERT can be regulated by the Myc/Max/Mad network protein on its promoter (58). Therefore, ViMIC provides information about this VIS integrating into the host genome which may be a functional element interacting with MYC. If users wish to observe whether mutations harbored in VIS ‘1000606’, they can click the ‘View VIS-VM association’ button to quickly search DVID in the ‘Mutation’ and ‘Sequence’ panel to acquire the association information. Moreover, users can also select ‘chr 5’ in the integration menu of HBV to inspect the overlap distribution of three factors and virus integration fragments on human chromosome 5. In addition, users can select the factor browse menu to view all overlapped entries between curated HBV VISs and MYC in the human genome (Figure 2B). All of the information would help users to provide clues to the interaction between viral integration and transcription factor binding in regulating target genes.

##### Case 3: visualization of target gene information and immune analysis result

As TERT is a target gene of ‘1000606’, ViMIC also presents the immune analysis between TERT gene expression and 22 types of infiltrating immune cells based on CIBERSORT (Figure 2C). The detailed information page of TERT shows the heatmap and correlation analysis results for HBV infected diseases as well as the TERT gene name, ID, full name, alias, transcripts, type, HPA location and protein classification information, and several gene ID links to other resources. The expression of TERT gene is related to the replication lifetime of CD8 (+) cytotoxic T lymphocytes (59). In ViMIC, the TERT gene expression analysis results further showed the positive correlation trend with CD8 + T cells in HBV + samples.

##### Case 4: HBV interactions with four histone marks

Previous studies have showed the key role of viral integration into the human genome and its potential to affect histone modification and transcription factor binding in human diseases. Bo Yang, *et al.* studied HBV interactions with human chromatins and investigated the significance of four histone modifications (H3K27ac, H3K9ac, H3K4me1 and H3K9me3) on HBV integration segment (60). They found that H3K27ac, H3K9ac and H3K4me1 were enriched in the region of viral integration on Chr2 and the region of VISs on Chr21 was enriched in H3K9me3, which indicated that HBV integration affected chromatin structure formation. The analysis of the VIS-cistrome significance (VIS-cistrome overlaps divided by peak numbers and sample counts) of all histone modifications in ViMIC in the same HBV insertion region mentioned by Bo Yang *et al.* was performed. The results are broadly consistent with Bo Yang *et al.* On Chr2, the results of H3K27ac, H3K9ac and H3K4me1 were nearly three to four times higher than that of H3K9me3, but on Chr21, the score of H3K9me3 achieved 5–10 times greater than those of H3K27ac, H3K9ac and H3K4me1 (Supplementary Figure S2). More importantly, ViMIC finds other potential histone modifications that may affect the formation of chromatin structure, such as active mark H3K4me3 and transcriptional elongation mark H3K36me3. Therefore, users can search and check the VIS of interest and inspect which cistrome data (histone mark, transcription factor and chromatin accessibility) overlapped this VIS. ViMIC may give users insights into the impact of viral integration on the host genome and exploring the potential mechanism with biology process of histone modification and transcription factor binding.

##### Case 5: evidences for essential mutants

The immune response plays a key role in the control of virus infection. Several studies have demonstrated that virus mutations allowing escape from the host immune response generally lead to the resistance to antiviral therapy and progression to disease (61,62). For example, in HBV genome, amino acid substitutions in the S region producing the mutated surface antigen may induce immune escape, such as G145R (63), Q129H (64) and I195M (65), etc. Moreover, immune-escape mutations may increase fitness of drug-resistant strains. sI195M, corresponding to rt the change of rtM204V, can raise the possibility of lamivudine-resistant and have the potential to immune escape (65). In ViMIC, users can easily view the mutation whether it is a reported escape/drug-resistant mutant, which will be of great benefit and convenience to researchers in the following situations: (i) the inquiry and summary of essential pathogenic mutations and (ii) providing supported evidences for the user to screen immune escape or drug resistance mutations on their own data.

## DISCUSSION AND FUTURE DIRECTIONS

Compared to our previous work on Dr. VIS, ViMIC has been developed as a comprehensive database of human disease-related viruses from the perspective of VMs, VISs and virus cis-effects, and is a well-maintained resource that will be of great value to many users.

ViMIC collected virus mutation and sequence information retrieved from a large collection of published literature. Previous studies have demonstrated that vital quasispecies features showed a promising clinical potential for disease prediction (25,26,66). This feature will not only provide researchers who are interested in VMs with annotations of the reported pathogenic viral mutations related to human diseases easily, but also may help spur the further investigation of potential sequence feature patterns, immune escape sites or drug resistance sites. ViMIC also integrate VIS data with cistrome resources. The viral sequence inserted into the host genome may be a functional element interacting with histone modification, transcriptional regulation and cis-regulatory elements. For example, Kelley *et al.* studied the interaction between HPV integration and histone marks in detail and found that enrichment regions of some histone marks correlate with HPV integration sites (67). Meanwhile, mutations in VISs may play an important role in virus infection and disease progress. Previous studies pointed out that HBx can enhance virus replication leading to a higher frequency of HBV–DNA integration events into the human genome (11). The HBx sequence easily integrates into hepatocytes with multiple point mutations which promotes the development of liver cancer (13). Recently, the study of Zheng *et al.* has used paired samples to draw the landscape of VISs and VMs in HBV-associated cirrhosis and hepatocellular carcinoma (28). Our work will contribute to and help researchers who are interested in gene regulation mechanisms to systematically investigate the functional annotation of VISs in the host genome. Furthermore, Joo *et al.* (68) indicated that the viral target genes related to an immune response may be associated with the viral integration patterns to avoid the immune surveillance system in the body and affect the response to treatment. ViMIC provides three aspects of viral target gene information for users to browse the gene information: VISs insertion information, and the correlation between the expression level of viral target genes and infiltrating immune cell fractions. Additionally, advanced users can conveniently download the annotated virus data and conduct further analysis by themselves.

In the future, ViMIC will be maintained and updated continuously, since VMs, VISs and cistrome data are expected to accumulate. New viruses can be added. The utility of ViMIC will be improved in several aspects. For example, the automatic recognition technology of mutations in the literature will be further optimized. The sequence around the VISs will be downloaded from public databases to perform in-depth analysis to investigate the functional role of virus-host interactions. The upload module will be optimized for users to upload potential VISs of interest in batches and perform online analysis. In addition, we will also integrate other immune deconvolution tools such as TIMER (69) and xCell (70), to improve the analysis of the association between gene expression and immune cell infiltration in the viral target gene module. In summary, we hope that ViMIC can give the biomedical community a better comprehensive understanding of virus-related research.

## DATA AVAILABILITY

ViMIC is available at http://bmtongji.cn/ViMIC/index.php. The E-Utilities is a set of eight server-side programs that provide a stable interface into the Entrez query and database system available in NCBI (https://www.ncbi.nlm.nih.gov/books/NBK25500/). GIGGLE can search and rank the overlapping genomic loci between query genomic locations such as VISs and genome interval files and it is available as a GitHub repository at https://github.com/ryanlayer/giggle. LiftOver is a web service that provides genome coordinate transformation between different human genome reference assemblies and can be freely accessed at https://genome.ucsc.edu/cgi-bin/hgLiftOver. PubTator is a web service that can automatically annotate biomedical entries including genes and mutations, from the literature, which is available at https://www.ncbi.nlm.nih.gov/research/pubtator/. CIBERSORT uses the gene expression data to estimate the abundances of member cell types from a mixed cell population, and it can be freely accessed at https://cibersort.stanford.edu/.

## Supplementary Material

gkab779_Supplemental_File
