# Supplementary material for: ViMIC: a database of human disease-related virus mutations, integration sites and cis-effects
Source: Nucleic Acids Res. 2021 Sep 9;50(D1):D918–27. doi: 10.1093/nar/gkab779 (PMC8728280; doi:10.1093/nar/gkab779)
Supplement: gkab779_Supplemental_File [file gkab779_supplemental_file.pdf]

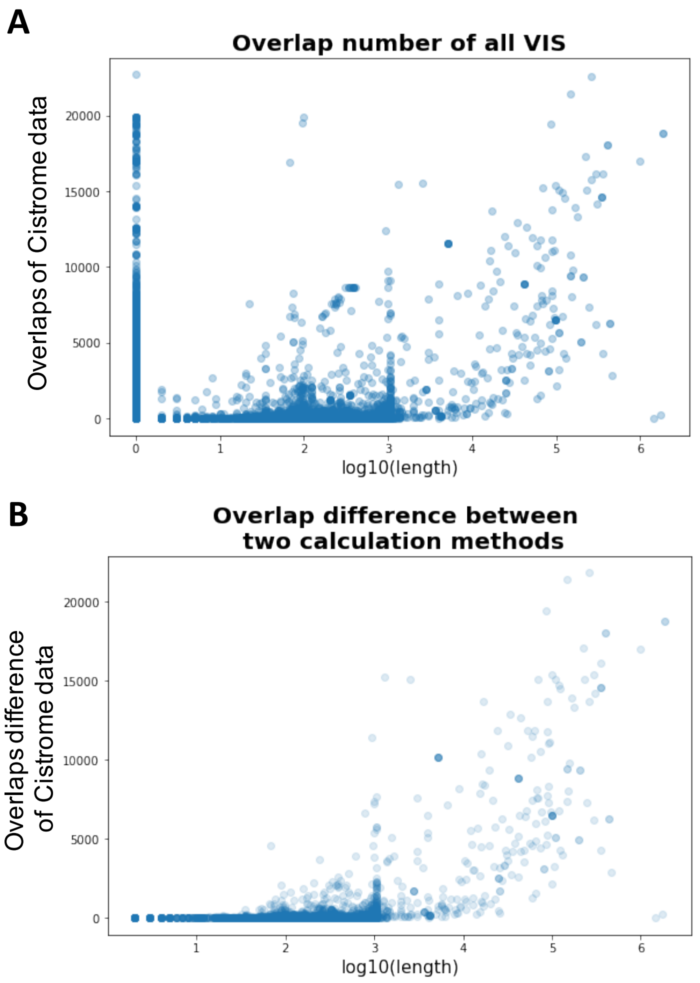

Figure S1. Analysis of the impact of the distance of two positions (length) on the overlap calculation

(A) Comparison of overlaps between VIS with single site and two positions. The X axis represents log10 (length), and the Y axis represents the total overlap of Cistrome data.

(B) Comparison of the overlap difference between VIS with single site and two positions. The X axis represents log10 (length), and the Y axis represents the overlap difference of Cistrome data.

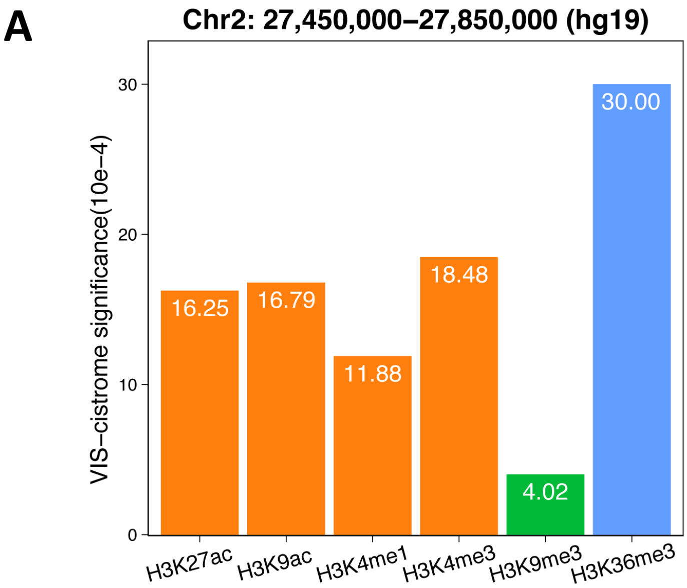

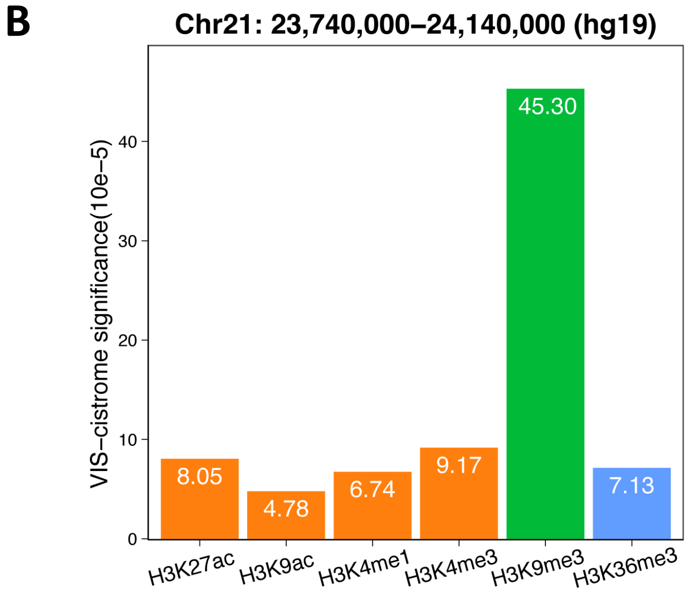

Figure S2. The significance score of six histone modifications on Chr2 and Chr21

The comparison of the VIS-cistrome significance of histone modifications in ViMIC in the same HBV insertion region mentioned by Bo Yang, et al. The VIS-cistrome significance referred to VIS-cistrome overlaps divided by peak numbers and sample counts. Active mark (orange): H3K27ac, H3K9ac, H3K4me1, H3K4me3; repressive mark (green): H3K9me3; transcriptional elongation (blue): H3K36me3. (A) Chr2: 27,450,000-27,850,000 (hg19) (B) Chr21: 23,740,000-24,140,000 (hg19)
